# Umbilical hernia repair and recurrence: need for a clinical trial?

**DOI:** 10.1186/s12893-021-01358-1

**Published:** 2021-10-12

**Authors:** Jennifer Mannion, Mohamed Khalid Hamed, Ritu Negi, Alison Johnston, Magda Bucholc, Michael Sugrue

**Affiliations:** 1 Department of Surgery, Letterkenny University Hospital, Donegal, Ireland; 2 Swami Rama Himalayan University, Himalayan Institute of Medical Sciences, Dehradun, India; 3 Emergency Surgery Outcome Advancement Project, Donegal Clinical Research Academy, Donegal, Ireland; 4 Intelligent Systems Research Centre, School of Computing, Engineering and Intelligent Systems, Ulster University, Londonderry, Northern Ireland

**Keywords:** Umbilical hernia repair, Open, Laparoscopic, Hernia recurrence, Mesh

## Abstract

**Introduction:**

Umbilical hernia repair, despite its perceived simplicity, is associated with recurrence between 2.7 and 27%, in mesh repair and non mesh repair respectively. Many factors are recognized contributors to recurrence however multiple defects in the linea alba, known to occur in up to 30% of patients, appear to have been overlooked by surgeons.

**Aims:**

This systematic review assessed reporting of second or multiple linea alba defects in patients undergoing umbilical hernia repair to establish if these anatomical variations could contribute to recurrence along with other potential factors.

**Methods:**

A systematic review of all published English language articles was undertaken using databases PubMed, Embase, Web of Science and Cochrane Library from January 2014 to 2019. The search terms ‘Umbilical hernia’ AND ‘repair’ AND ‘recurrence’ were used across all databases. Analysis was specified in advance to avoid selection bias, was registered with PROSPERO (154173) and adhered to PRISMA statement.

**Results:**

Six hundred and forty-six initial papers were refined to 10 following article review and grading. The presence of multiple linea alba defects as a contributor to recurrence was not reported in the literature. One paper mentioned the exclusion of six participants from their study due multiple defects. In all 11 factors were significantly associated with umbilical hernia recurrence. These included: large defect, primary closure without mesh, high BMI in 5/10 publications; smoking, diabetes mellitus, surgical site Infection (SSI) and concurrent hernia in 3/10. In addition, the type of mesh, advanced age, liver disease and non-closure of the defect were identified in individual papers.

**Conclusion:**

This study identified many factors already known to contribute to umbilical hernia recurrence in adults, but the existence of multiple defects in the linea, despite it prevalence, has evaded investigators. Surgeons need to be consider documentation of this potential confounder which may contribute to recurrence.

**Supplementary Information:**

The online version contains supplementary material available at 10.1186/s12893-021-01358-1.

## Introduction

**Fig. 1:**
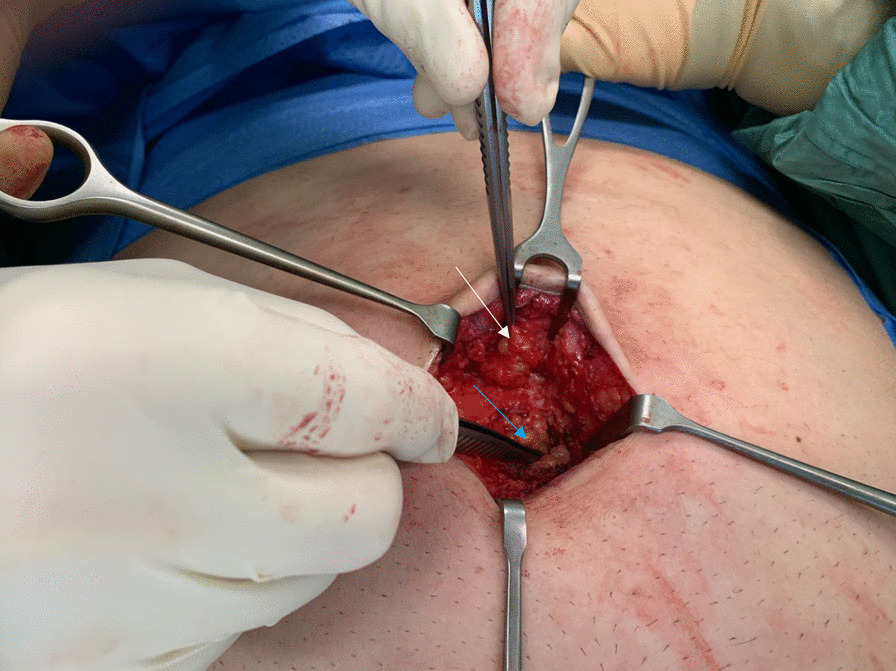
Picture showing fenestrated linea alba with a hernia protruding through second defect (white arrow) cranial to the primary defect (blue arrow)

While umbilical hernia (UH) repair is often considered a “simple” operation there has been some controversy regarding the preferred technique between primary and mesh augmented repair. This resulted from data showing the once preferred Mayo repair had a recurrence rate of up to 50% [1]. It became apparent that the use of mesh augmentation of a repair, whether inlay, sublay or onlay, added additional benefit [2, 3]. In 2010 Sugrue et al. [4] identified the frequent presence of multiple fascial defects in close proximity to the main umbilical hernia and coined the term “Fenestrated Linea Alba” (Fig. 1). This supplemented previous work by Moschowitz one century earlier who identified lacunar defects in the linea alba [5, 6]. These two reports are limited in their scope and the latter is of historic interest.

Despite international guidelines [7] suggesting that a mesh is mandatory when repairing UH, particularly in large defects, the recurrence rate in the most recent literature ranges from 2.7 to 10% [8]. Given the reported frequency of the fenestrated linea alba could this be a contributor to recurrence? Many factors associated with recurrence are already well known [9]. This study undertook a systematic review to analyse the reporting of second defects during UH and whether they may potentially contribute to recurrence.

## Methods

A systematic review of all published English language articles was undertaken in Letterkenny University Hospital in October 2019 using the electronic databases PubMed, Embase, Web of Science and Cochrane Library over a 5 period from January 2014 to September 2019. The reproducible search strategy ‘Umbilical hernia AND repair’ ‘open’ ‘laparoscopic’ AND ‘recurrence’ were used across all databases to include relevant papers.

### Eligibility assessment and data extraction

The primary outcome was the documentation of second or multiple defects in the linea alba adjacent to the primary hernia in patients undergoing primary or recurrent UH repair.

The method of analysis and inclusion were specified in advance to avoid selection bias and documented in a protocol, which was registered with the International Prospective Register of Systematic Reviews (CRD4202154173). This systematic Review adhered to the Preferred Reporting Items for Systematic Reviews and Meta-Analyses (PRISMA) statement [10].

Studies were included in the systematic review if the following criteria were met: either open, laparoscopic or robotic UH repair with reporting of the intraoperative findings relating to the nature of the defect and/or where factors contributing to recurrence were reported. Studies based on paediatric, pregnant or cirrhotic patients or those with inguinal or complex ventral hernias were not included. Reviews, meta-analyses, case reports, errata, letters, protocols, surveys, abstracts, non-English language and studies that did not report outcomes and those with inadequate data to allow interpretation, were not included.

Eligibility assessment was performed independently in a blinded standardised manner by two reviews (JM, MH) and compared to ensure data extraction was complete. Disagreement between reviewers was resolved by third author review (RN).

The descriptive data from the screened studies was extracted by two reviewers (JM, MH) and compared to ensure extraction was complete. Data was collected using a data extraction sheet.

### Quality assessment

The Methodological Index for Non-Randomised Studies (MINORS) criteria [11], was used for quality assessment of comparative and non-comparative surgical studies using a 3-point scale (0 not reported, 1 reported but inadequate, 2 reported and adequate) on eight items for non-comparative studies and 12 items for comparative studies. The ideal global score chosen for inclusion in this study was at 10 for non-comparative and 15 for comparative studies. Three reviews (JM, MH, RN) performed quality assessment independently in a blinded standardised manner. Disagreements were resolved by discussion between the review authors and if agreement could not be reached then by a fourth reviewer (AJ).

## Results

**Fig. 2:**
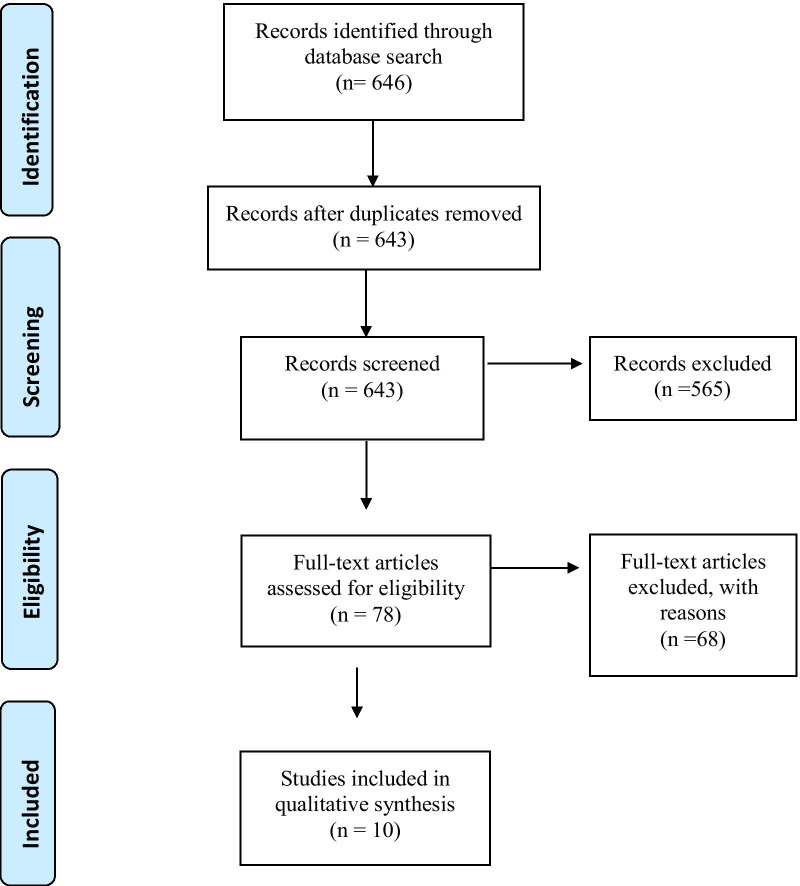
Identification, review and selection of articles included in the systematic review, shown by PRISMA flow chart

This study reviewed 646 articles of which 10 were found to meet the inclusion criteria as shown in the PRISMA flow chart, Fig. 2.

The presence of multiple linea alba defects was not reported in any of the papers however Kauffman in a randomized control trial of 300 patients undergoing UH repair described in their methodology that they excluded 6 patients from their study due to them having multiple defects [12].

There were 17 factors reported to influence recurrence rates as outlined in Table 1.

**Table 1:** Contributors to recurrence rate (RR) in umbilical hernia repair

Author	Study design and (n = 4363)	Contributing factor	Conclusion
Christofferson (2015)	Cohort Study N = 1313	Use of mesh versus primary repair	Overall RR 10% with mesh and 21% for primary suture repair (p = 0.001)
Cheng (2018)	Cohort Study N = 168	use of a mesh ventral patch	RR 2.4% and SSI rate of 4.7%. When Intraperitoneal placement of mesh performed SSI was 19%
Donovan (2019)	Cohort Study N = 979	Age, sex, body mass index, concurrent laparoscopic inguinal hernia repair, smoking status, diabetes, postoperative infection, hernia size, type of repair	RR of 3.3%. Higher BMI (p = 0.007), concurrent laparoscopic inguinal hernia repair (p = 0.044), current smoking (p = 0.020), diabetes (p = 0.021), and primary closure repair of hernias > 1.5 cm (p = 0.001) had a greater risk of recurrence
Froylich (2016)	Cohort study N = 186	Laparoscopic versus open repair	RR in the laparoscopic was 20% vs. 27.1% for open (p = 0.28)
Kauffman (2018)	Randomized Control Trial N = 300	Use of Mesh versus Primary Repair	RR in mesh 3·6% [95% CI 1·4–9·4] *vs* 11·4% (6·8–18·9) in suture repair (p = 0.01)
Mitura (2017)	Cohort Study N = 82	Closure of defect (IPOM plus) versus bridging mesh (IPOM)	IPOM plus had no recurrence vs. 10% RR for standard IPOM (p = 0.018)
Ponten (2019)	Randomized control trial = 352	Mesh ventral patch (PVP) versus standard prolene mesh	No significant differences were seen in RR (n = 13, 8.4% PVP vs n = 6, 4.1% mesh (p = 0.127)
Shankar (2017)	Cohort study N = 332	Use Mesh versus Primary repair and multiple other demographic factors	Ascites, liver disease, diabetes, obesity, and primary suture repair were significantly with RR. Primary suture RR 9.8% vs. 2.4% in mesh (p = 0.04)
Winsnes (2016)	Cohort Study N = 306	Use of Mesh versus Primary Repair	RR of 8.4% (8% mesh v 9% suture OR 0.90, 95% CI 0.40–2.02) Complication rate was significantly higher in patients receiving mesh repair OR 6.63, 95% CI 2.29–20.38. Coexisting hernia OR 2.84, 95% CI 1.24–6.48
Yao (2016)	Cohort Study N = 199	Primary Suture repair in obese patients	RR obese vs. non-obese not significantly different 3.7% vs 4.6%, (p = 5.72). BMI no association with complications

The incorporation of mesh augmentation into the field of hernia surgery has become widespread, the effect of mesh on recurrence rates and its utilization based on the size of the defect was discussed in 6 papers. Christoffersen in a study of 1313 patients described an overall recurrence rate of 18% for small umbilical hernias < 2 cm. Mesh repair had a significantly lower recurrence rate of 12% compared to 21% in primary repair. At an average of 55 months follow up the recurrence rate was 21% for primary repair and 10% for mesh repair p = 0.001[13]. Kaufmann in their study of 300 patients, reported a cumulative reduction in recurrence from 11.4 to 3.6% when mesh was used. Subgroup analysis of 1–2 cm and 2–4 cm defects showed that irrespective of size, mesh augmentation had lower recurrence rates [12]. Shankar in their study of 332 participants report an overall recurrence rate of 6% and found no difference based on the size of the defect [14].

Iatrogenic enlargement of small defects to allow for mesh placement was not found on subgroup analysis to be associated with an increased recurrence [15]. Donovan in their cohort study of 979 patients reported no difference in recurrence rates between hernia size groups ranging between < 1 cm, 1–1.4 cm, 1.5–1.9 cm, 2–2.4 cm p = 0.957. However they reported that in defects > 1.5 cm repaired with primary closure, the recurrence rate was 7.3% p = 0.0442, which on multivariate analysis was seven times greater than when mesh was used HR 6.79 [2.20–20.92] [16].

On the other hand, Winsnes not only found that there was no difference in recurrence if a mesh were used, but the complication rate increased significantly [17]. A failing of these mesh/non-mesh studies is the lack of documentation of the presence or absence of multiple defects.

The effect of body mass index (BMI) on recurrence was evaluated in 5 studies with inconclusive causal association to recurrence. A retrospective cohort study of 332 patients found that on multivariate analysis obesity is associated with increased risk of recurrence (OR 3.3 CI 95% 1.0–10.1) [14]. A prospective cohort study of 1125 patients found no statistical difference between BMI subgroups on univariate analysis (p = 0.057) however on multivariate analysis every 1 kg/m^2^ in BMI increase was associated with about a 9% increased risk of recurrence (p = 0.0072) [16]. Elevated BMI in a cohort study of 306 patients on univariate analysis reported an increased risk for recurrence (OR 2.23 CI 1.17–4.26) however on subsequent multivariate analysis this was no longer significant [17]. Multicentre randomized trial of 300 patients reported no difference in recurrence rates between BMI subgroups [12]. A retrospective review of 199 patients undergoing primary tissue repair found an overall recurrence rate of 4% with no difference in obese patients on subgroup analysis [18].

The effect of smoking was analysed in 3 studies. A prospective cohort study found that there was no significant increase in recurrence in smokers (OR 1.01 95% CI 0.29–3.56) [17]. A more recently published prospective cohort study contradicts this and reports recurrence rates of 3% in non-smokers compared to 8% in smokers (p = 0.02) [16]. A multicentre observational cohort study of 168 patients undergoing ventral hernia repair with mesh, of which 65% were umbilical defects, reported a recurrence rate of 2.4% and that smoking was the only significant factor to increase recurrence (p = 0.022)[19].

Concurrent repair of co-existing ventral or inguinal herniae was reported in 3 studies all of which found it elevated the risk of recurrence [14, 17]. The largest study reported that the presence of co-existing inguinal hernia was statistically significant with a HR 2.54 (CI 95% 1.03–6.27 p = 0.0437)[16].

Diabetes Mellitus was identified in 3 studies with two finding diabetic patients had an increased risk of recurrence with Donovan reporting 7.8% recurrence in diabetics compared to 2.8% for non-diabetics p = 0.02 [14, 16]. However a cohort study of 306 patients found no association between diabetes and recurrence OR 0.3 (CI 0.04–2.28) [17].

The effect of Surgical Site Infection (SSI) was reported in 3 studies however it was not statistically significant in influencing recurrence [15, 16, 18].

The influence of mesh type was analysed in 2 studies. Ponten reported no difference in recurrence between the use of a ventral patch (8.4%) when compared to a standard prolene mesh (4.1%) p = 0.127 [15]. Donovan reported on the use of various mesh types including polytetrafluoroethylene, polypropylene, polydiaxone, polyester and multifilament polypropylene in open UH repair and found no statistical difference in recurrence [16].

The presence of liver disease and ascites was identified as a factor for recurrence in a study of 332 patients by Shankar who reported an OR 8.0 (CI 95% 1.8–34.4 p = 0.02 [14].

In a retrospective review of 186 obese patients, Froylich reports a recurrence rate of 20% in laparoscopic repair versus 27% (p = 0.28) in open ventral hernia repair at 6 years follow up; the study identified that advanced age was a protective factor with an OR − 0.03 (CI 0.96–0.01 p = 0.01)[20].

Closure of fascial defect in laparoscopic ventral hernia repair, termed ‘IPOM Plus’, was assessed by Mitura in their case–control trial of 82 patients. Recurrence rates were significantly decreased from 10% in standard IPOM down to 0% when fascial closure was performed [21].

American Society of Anaesthesiologist (ASA) score was reported as a factor is recurrence rates with patients having an ASA III/IV being more likely to experience recurrence on multivariate analysis [16].

One paper reports on univariate analysis that Chronic Kidney Disease, Cardiac disease and presence of bowel obstruction on presentation are all factors that increase recurrence [18].

## Discussion

This systematic review demonstrates that the presence of multiple defects in patients undergoing UH repair is not being reported in the scientific literature. This is surprising given Moschcowitz’s original description of multiple small defects in the linea alba, in what he coined the lacunar theory of perforation blood vessels from the pre-peritoneal space [5]. Sugrue in a personal series of 146 open UH repairs found that 25% had a second defect. There were more than 2 defects in 18% of his series and he coined the term fenestrated linea alba [4]. These two papers however provide no evidence that fascial defects are a contributor to umbilical hernia.

The introduction of videoscopy has expanded our knowledge of hernia anatomy and it has been reported that in a prospective study of 146 patients undergoing laparoscopic repair of ventral hernia, 50% had occult defects. These defects were unappreciable during preoperative clinical examination [22]. Occasionally a second defect is even visible in or adjacent to the umbilical ring on clinical examination (See Additional file 1). While a hernia arising from these defects does not represent a true recurrence but rather an unappreciated second defect at the time of surgery, they present clinically as recurrences of the primary hernia repair and so will be classified and recorded as recurrence in the literature. The role of these occult secondary defects in recurrence is unclear and future clinical trial assessing the operative strategy should include documentation of the presence or absence of a fenestrated linea alba or second defect. Cranial dissection for 2–5 cm along the linea alba, and their subsequent repair of identified additional defects may prevent recurrence.

While UH repair is a common procedure it is really only in the last 2 decades that predictors of recurrence are increasingly understood. This review also sought to identify the key factors that were reported which may be responsible for increased recurrence. The use of primary repair rather than mesh augmentation appears to be a factor in recurrence although this was not necessarily significant when used in smaller umbilical hernias < 1.5 cm. The use of mesh is not without risk and increasingly there are reports that mesh augmentation is associated with an increased complication profile which has led to medicolegal disputes [23]. This study found conflicting data to support this and while some included studies reported wound infections occurring more frequently with mesh augmentation, this was not true for all studies, although it is worth noting that such complications were not necessarily an endpoint specifically measured by some of the studies. Patient related factors such as a diagnosis of Diabetes Mellitus and having a concurrent hernia were found to significantly increase risk of recurrence in the majority of the studies and this certainly supports the hypothesis that abnormal collagen synthesis is both a factor in hernia formation and post operative recurrence [24]. The hypothesis of ‘field defects’ secondary to collagen disorder has been described and may further contribute to our understanding of why these secondary defects occur [14]. Rectus Diastasis while associated with gradual thinning and widening of the linea alba, is not in a true hernia, as there is musculofascial continuity and absence of a hernia sac. Rectus Diastasis was not identified as a contributor to recurrence in this systematic review, however a retrospective cohort study by Köhler in 2015 reported that 45% of patients undergoing umbilical hernia repair had concomitant rectus diastasis and proposes that deterioration in the connective tissue causing stretching may also be a risk factor for midline hernia formation and recurrence [25, 26]. For obesity, the data is less conclusive, with the larger randomized trial finding no significance however the follow up period of 25 months was substantially shorter than the cohort studies which reported up to 8.5 years follow up. The incidence of surgical site infection was reported in three studies but only significant in one which is contradictory to recent evidence from ventral and incisional hernia repair where patients with infection had double the risk of recurrence [27].

It is important to interpret the reported results with caution as the data is quite heterogeneous and predominantly from retrospective cohort studies. The sample sizes varied greatly between studies and the definition of given variables such as obesity were not congruent as it was used as both a categorical and a continuous variable in the various studies. The follow up period ranged from 14 months to 8.5 years, with none of them reaching the recommended 10 year follow up [28]. Another major limitation was statistical underpowering, particularly when multivariate analysis was performed, which challenges the ability to reach a scientifically sound conclusion [14]. Unfortunately, due to the heterogeneous nature of the data reported, meta-analysis was not possible.

## Conclusion

UH repair is increasingly evaluated, with a focus on outcomes, allowing the identification of unacceptably high recurrence rates. Surgical operative assessment should look for multiple defects and factor their repair into the procedure. While the use of mesh, the size of the defect and patient characteristics such as BMI and Diabetes Mellitus are recognised in the literature as contributors to recurrence, the role of the fenestrated linea alba in contributing to perceived umbilical hernia recurrence appears to be overlooked and its role should be included in further studies to enhance our ability to reduce recurrence.

## Supplementary Information

**Additional file 1: Video 1.** Demonstrating a secondary defect in the linea alba during open umbilical hernia repair. **Video S2.** Showing secondary defect on clinical examination

## Data Availability

The datasets used and/or analysed during the current study available from the corresponding author on reasonable request.

## References

[1] Paul A, Korenkov M, Peters S, Köhler L, Fischer S, Troidl H (1998). Unacceptable results of the Mayo procedure for repair of abdominal incisional hernias. Eur J Surg.

[2] Aslani N, Brown CJ (2010). Does mesh offer an advantage over tissue in the open repair of umbilical hernias? A systematic review and meta-analysis. Hernia.

[3] Bittner R, Bingener-Casey J, Dietz U, Fabian M, Ferzli GS, Fortelny RH, Köckerling F, Kukleta J, Leblanc K, Lomanto D (2014). Guidelines for laparoscopic treatment of ventral and incisional abdominal wall hernias (International Endohernia Society (IEHS)-part 1. Surg Endosc.

[4] Sugrue ME (2010). Fenestrated linea alba a potential trap in umbilical hernia repair- beware!. Ir J Med Sci.

[5] Moschcowitz AV (1914). The pathogenesis and treatment of hernise of the linea alba. Surg Gynec Obst.

[6] Moschcowitz AV (1915). The pathogenesis of umbilical hernia. Ann Surg.

[7] Heniford BT (2016). SAGES guidelines for laparoscopic ventral hernia repair. Surg Endosc.

[8] Ra M (2019). Umbilical hernia: when and how. Ann Laparosc Endosc Surg.

[9] Venclauskas L, Jokubauskas M, Zilinskas J, Zviniene K, Kiudelis M (2017). Long-term follow-up results of umbilical hernia repair. Wideochir Inne Tech Maloinwazyjne.

[10] Moher D, Liberati A, Tetzlaff J, Altman DG (2009). Preferred reporting items for systematic reviews and meta-analyses: the PRISMA statement. PLoS Med.

[11] Slim K, Nini E, Forestier D, Kwiatkowski F, Panis Y, Chipponi J (2003). Methodological index for non-randomized studies (minors): development and validation of a new instrument. ANZ J Surg.

[12] Kaufmann R, Halm JA, Eker HH, Klitsie PJ, Nieuwenhuizen J, Van Geldere D, Simons MP, Van der Harst E, van’t Riet M, Van der Holt B (2018). Mesh versus suture repair of umbilical hernia in adults: a randomised, double-blind, controlled, multicentre trial. Lancet.

[13] Christoffersen MW, Helgstrand F, Rosenberg J, Kehlet H, Strandfelt P, Bisgaard T (2015). Long-term recurrence and chronic pain after repair for small umbilical or epigastric hernias: a regional cohort study. Am J Surg.

[14] Shankar DA, Itani KMF, O'Brien WJ, Sanchez VM (2017). Factors associated with long-term outcomes of umbilical hernia repair. JAMA Surg.

[15] Ponten JEH, Leclercq WKG, Lettinga T, Heemskerk J, Konsten JLM, Bouvy ND, Nienhuijs SW (2019). Mesh OR patch for hernia on epigastric and umbilical sites (MORPHEUS-Trial): the complete two-year follow-up. Ann Surg.

[16] Donovan K, Denham M, Kuchta K, Denham W, Linn JG, Haggerty SP, Carbray J, Ujiki M (2019). Predictors for recurrence after open umbilical hernia repair in 979 patients. Surgery.

[17] Winsnes A, Haapamäki MM, Gunnarsson U, Strigård K (2016). Surgical outcome of mesh and suture repair in primary umbilical hernia: postoperative complications and recurrence. Hernia.

[18] Yao JJ, Pham T, El Mokdad A, Huerta S (2016). Predictors of recurrence of umbilical hernias following primary tissue repair in obese veterans. Am J Surg.

[19] Cheng D, Bonato L, Leinkram C (2018). Infection and recurrence rates of the C-QUR V-patch. Hernia.

[20] Froylich D, Segal M, Weinstein A, Hatib K, Shiloni E, Hazzan D (2016). Laparoscopic versus open ventral hernia repair in obese patients: a long-term follow-up. Surg Endosc.

[21] Mitura K, Skolimowska-Rzewuska M, Garnysz K (2017). Outcomes of bridging versus mesh augmentation in laparoscopic repair of small and medium midline ventral hernias. Surg Endosc.

[22] Saber AA, Rao AJ, Itawi EA, Elgamal MH, Martinez RL (2008). Occult ventral hernia defects: a common finding during laparoscopic ventral hernia repair. Am J Surg.

[23] Bittner R (2019). Medico-legal implications in hernia surgery. Int J Abdom Wall Hernia Surg.

[24] Zöller B, Ji J, Sundquist J, Sundquist K (2013). Shared and nonshared familial susceptibility to surgically treated inguinal hernia, femoral hernia, incisional hernia, epigastric hernia, and umbilical hernia. J Am Coll Surg.

[25] Köhler G, Luketina RR, Emmanuel K (2015). Sutured repair of primary small umbilical and epigastric hernias: concomitant rectus diastasis is a significant risk factor for recurrence. World J Surg.

[26] Reinpold W, Köckerling F, Bittner R, Conze J, Fortelny R, Koch A, Kukleta J, Kuthe A, Lorenz R, Stechemesser B (2019). Classification of Rectus Diastasis-A Proposal by the German Hernia Society (DHG) and the International Endohernia Society (IEHS). Front Surg.

[27] Jolissaint JS, Dieffenbach BV, Tsai TC, Pernar LI, Shoji BT, Ashley SW, Tavakkoli A (2020). Surgical site occurrences, not body mass index, increase the long-term risk of ventral hernia recurrence. Surgery.

[28] Köckerling F, Koch A, Lorenz R, Schug-Pass C, Stechemesser B, Reinpold W (2015). How long do we need to follow-up our hernia patients to find the real recurrence rate?. Front Surg.

