# Supplementary material for: Umbilical hernia repair and recurrence: need for a clinical trial?
Source: BMC Surg. 2021 Oct 12;21:365. doi: 10.1186/s12893-021-01358-1 (PMC8507103; doi:10.1186/s12893-021-01358-1)
Supplement: Supplementary file 1 — Additional file 1: Video 1. Demonstrating a secondary defect in the linea alba during open umbilical hernia repair. Video S2. Showing secondary defect on clinical examination [file 12893_2021_1358_MOESM1_ESM.docx]

**Supplementary video 1**. Demonstrating a secondary defect in the linea alba during open umbilical hernia repair


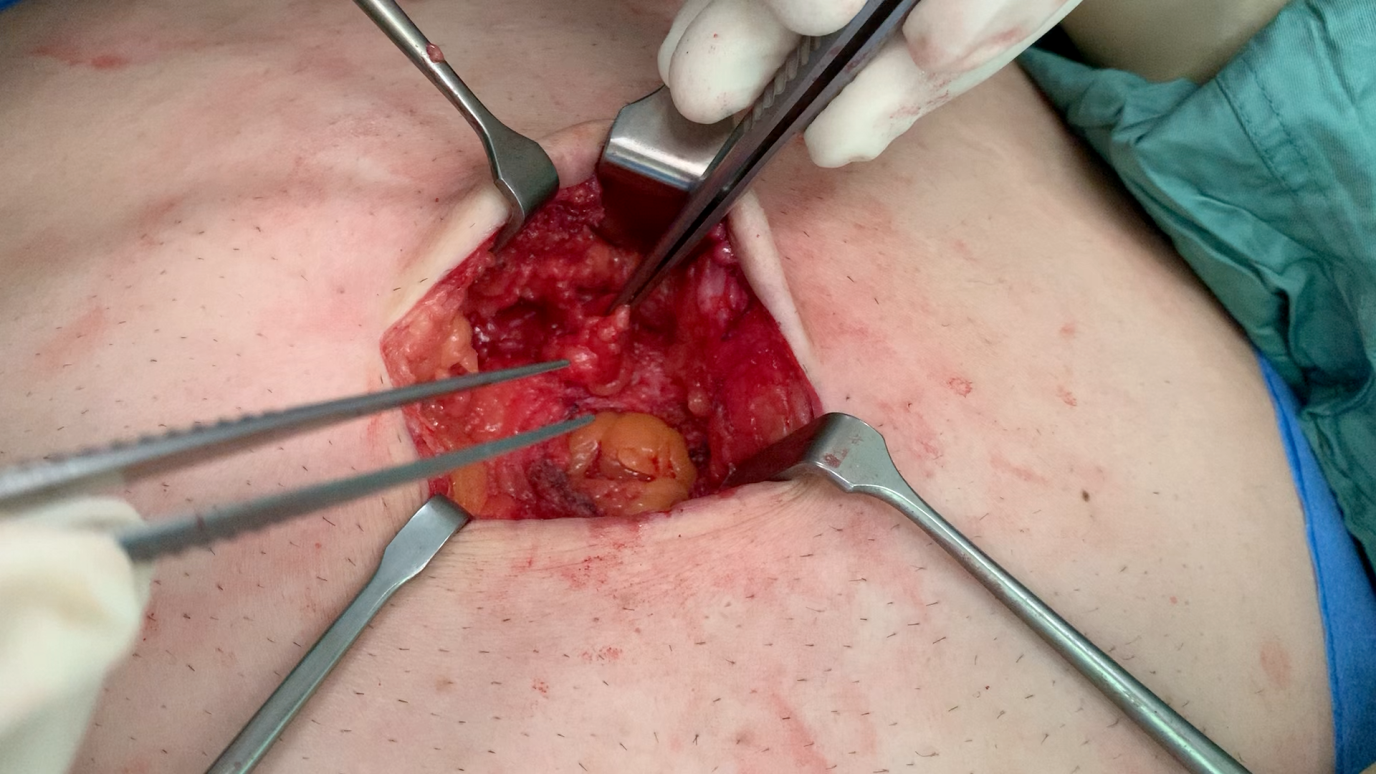


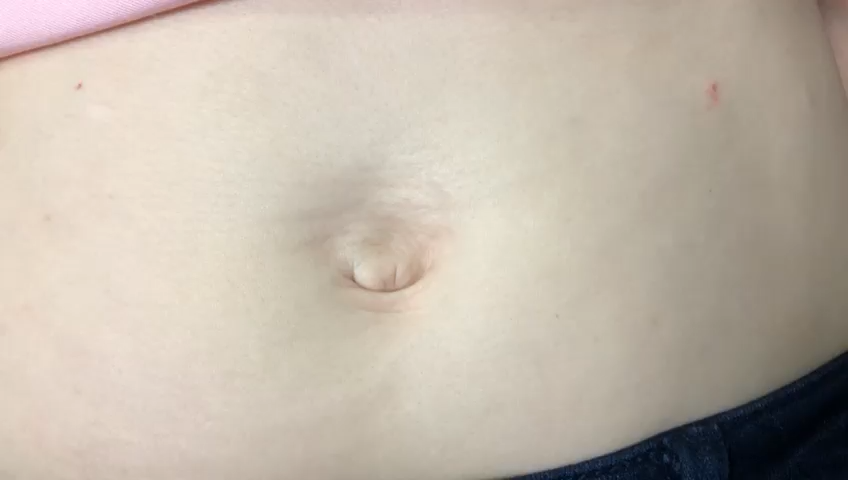
**Supplementary Video 2.** Showing secondary defect on clinical examination
